# Plant long noncoding RNAs: why do we not know more?

**DOI:** 10.1186/s40659-025-00610-9

**Published:** 2025-06-10

**Authors:** Paulina Kościelniak, Łukasz Walas, Agata Konecka, Włodzimierz Buraczyk, Ewelina A. Klupczyńska

**Affiliations:** 1 Institute of Human Biology and Evolution, Faculty of Biology, Adam Mickiewicz University, Uniwersytetu Poznańskiego 6, 61614 Poznań, Poland; 2 Institute of Dendrology, Polish Academy of Sciences, Parkowa 5, 62030 Kórnik, Poland; 3 Warsaw University of Life Sciences, Nowoursynowska 166, 02787 Warsaw, Poland

**Keywords:** lncRNA, miRNA, Plants, Genome size, Polyploidization, Genome duplication, Epitranscriptome, Coexpression, Species range, Computational analyses

## Abstract

Analysis of plant and animal genomes is essential for understanding their biological function, adaptation, and evolution. Human genomic databases are the most advanced due to extensive research on the genetic basis of disease and personalized medicine. Key resources include GenBank, Ensembl, the 1000 Genomes Project, and GTEx, which provide detailed information on genome sequences, genetic variation, and gene expression in different tissues. Similarly, genomic and transcriptome databases for animals are relatively well-developed, particularly for model organisms such as *Mus musculus*, *Drosophila melanogaster*, and *Danio rerio*. In contrast, plant genomic databases are developing rapidly but remain less comprehensive than those for humans and animals. This discrepancy is primarily due to the high species diversity and complexity of plant genomes, which are often characterized by gene duplication and significant structural variability. Databases such as Phytozome, TAIR (The Arabidopsis Information Resource), Gramene, and Planteome focus mainly on model plants and agriculturally important species. Another crucial factor is the lower funding for plant-related projects, despite the substantial investment required due to the large size and complexity of plant genomes. This disparity is also evident in the study of long non-coding RNAs (lncRNAs), which play a key role in the growth and development of organisms. In plants, genome complexity—driven by factors such as considerable length, polyploidy, and epigenetic modifications—poses significant challenges for research. Despite these obstacles, understanding lncRNAs in plants, particularly in forest trees, is of paramount importance. lncRNAs hold great potential for applications in agriculture and forestry, especially in the context of climate change. For example, they could enhance our ability to develop resilient tree species capable of withstanding environmental stressors. To achieve this, a comprehensive understanding of lncRNA functions at the molecular and biological levels, as well as the development of robust and complete databases, is urgently needed. In the near future, computational analyses are expected to play a key role in overcoming these challenges. In this article, we review the current state of knowledge about lncRNAs in plants, highlight the obstacles to their study, and explore how advances in this field could revolutionize agriculture and forestry. By focusing on the unique challenges and opportunities presented by forest trees, we emphasize the crucial role of lncRNA research in addressing global environmental challenges.

## Introduction

There is growing evidence that long noncoding RNAs (lncRNAs) are extensively involved in many cellular processes in humans and animals. Additionally, a significant body of research demonstrated that lncRNAs also play crucial roles in various stage of plant development, its enhance our understanding of the the mechanisms underlying lncRNA regulation and to develop comprehensive lncRNA databases [1]. Through the creation of detailed annotations, fundamental questions regarding lncRNA function can be addressed. Given this context, one wonders what are the main obstacles limiting lncRNA research in plants and are these perhaps problems that will soon, with the development of bioinformatics and data science, be largely overcome?.

**Fig. 1:**
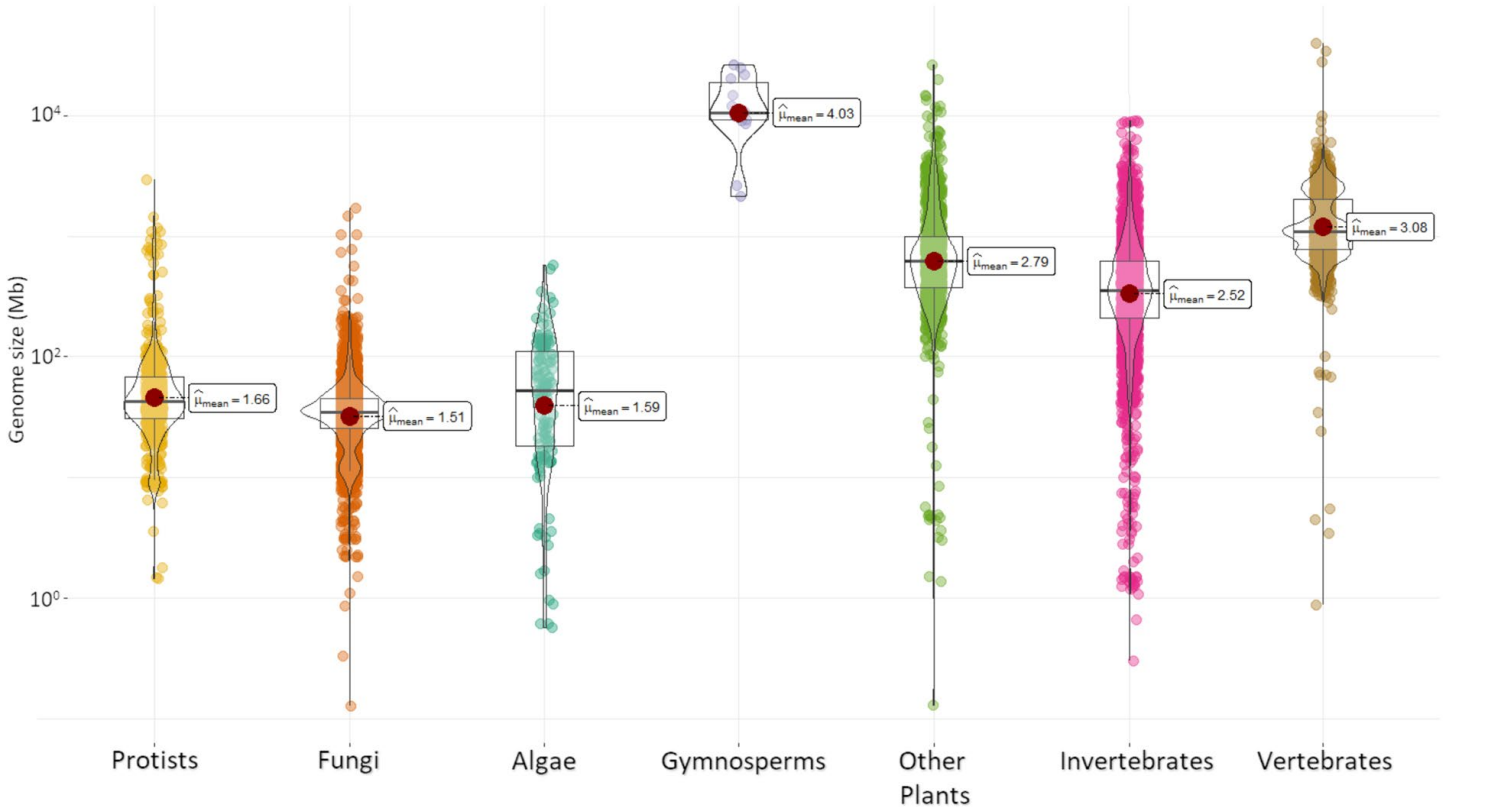
Genome size (logarithmic scale) in different taxonomic groups according to the NCBI data (only ;‘reference’’ and ‘‘representative’’ genomes). The data were visualized with the ‘ggstatsplot’ package [30]. Most animals, especially mammals, have similar-sized genomes (3–7 Gbp). However, in plants, the sizes of the genomes vary much more between species, reaching very large values (1–148 Gbp). Tree genomes typically contain tens of billions of base pairs (10–30 Gbp)

Comparative analysis of plant and animal genomes is vital for understanding their biological functions, adaptations and evolution. While databases containing animal and human data are far more advanced and precise than those for plants, the complexity of plant genomes presents unique challenges, as already mentioned. However, this topic deserves to be developed further, as the originality and individuality of plants is a very interesting evolutionary phenomenon [2, 3]. The considerable length of plant genomes (Fig. 1), along with the prevalence of polyploidy and epigenetic modifications, significantly complicates the acquisition of complete knowledge about these genomes. These factors have a profound impact on genome architecture, thereby increasing the complexity of understanding both coding and noncoding functions. Moreover, variations in genome length are closely tied to evolutionary history as mentioned, also to functional diversity, genetic structure, and, critically, the adaptation of sessile organisms like plants to their environments [4–6].

This focus on plant and animal genome analysis was selected due to its relevance in elucidating biological functions, adaptation, and evolution. A deeper exploration of long noncoding RNAs (lncRNAs), which are involved in numerous essential process during organism development, is particularly important. In contrast to animals, where databases are well-developed and accurate, research on plant genomes faces challenges. Advancing our understanding of genomic processes is critical for the creation of robust lncRNA databases, which are of great significance for field such as agriculture and forestry. 

## Genome architecture: genome size, polyploidization, C and G paradox value and epitranscriptom

### Genome size and polyploidization

One phenomenon that plays a crucial role in the evolution and adaptation of plants to different environmental conditions is genome duplication, which results in the formation of polyploids. Polyploidy leads to an increase in the amount of DNA in the genome, affecting its complexity, chromosome structure, and stability. Genome duplication drives genomic diversity and may contribute to short-term responses to enviromental stimuli [7]. At the biological level, polyploidy in plants may provide adaptive benefits, such as increased stress resistance, greater phenotypic plasticity and adaptability to varying environmental conditions [8–10]. Whole-genome duplications (WGDs) can also enable the emergence of new genes and functions involved in developmental processes, metabolism, stress responses and other biological functions or adaptation [11]. In contrast to classical gene duplication mechanisms, such as segmental duplications or whole-genome duplications, retrogens are formed in a process mediated by retrotransposons or cellular reverse transcriptase activity. Once integrated into the genome, retrogens can acquire new regulatory elements, leading to functional diversification [12]. In plants, retrogenes contribute to genomic innovation by introducing new gene variants that can undergo neofunctionalization, subfunctionalization, or even lead to the development of lineage-specific adaptations. Several studies have demonstrated that retrogenes play a role in stress responses, reproductive mechanisms, and metabolic pathway diversification [13]. For instance, in Arabidopsis thaliana and *Oryza sativa*, numerous retrogenes have been identified, some of which have acquired novel regulatory elements that contribute to stress adaptation and environmental responsiveness [14].

Retrogenes receive considerably more research funding in human and animal studies than in plant research, largely due to their biomedical implications. In humans and animals, retrogenes have been implicated in gene regulation and are associated with diseases such as cancer and neurodegenerative disorders, drawing substantial investment in pharmacological and medical research. In contrast, plant genomic funding is predominantly directed toward improving agricultural traits—like yield enhancement and stress resistance—while basic investigations into retroposition and retrogene functionality remain underfunded. Additionally, the inherent challenges of plant research, including complex genome structures and long life cycles, further hinder extensive functional analyses [12–14].

Compared to animal genomes, plant retrogenes exhibit a lower frequency due to structural constraints, including extensive genome duplications and frequent polyploidization events that shape plant genome evolution. However, their role in genetic novelty and adaptability remains significant [13]. The functional integration of retrogenes into regulatory networks demonstrates their importance in plant genome evolution, particularly in response to environmental pressures and selective forces.

**Fig. 2:**
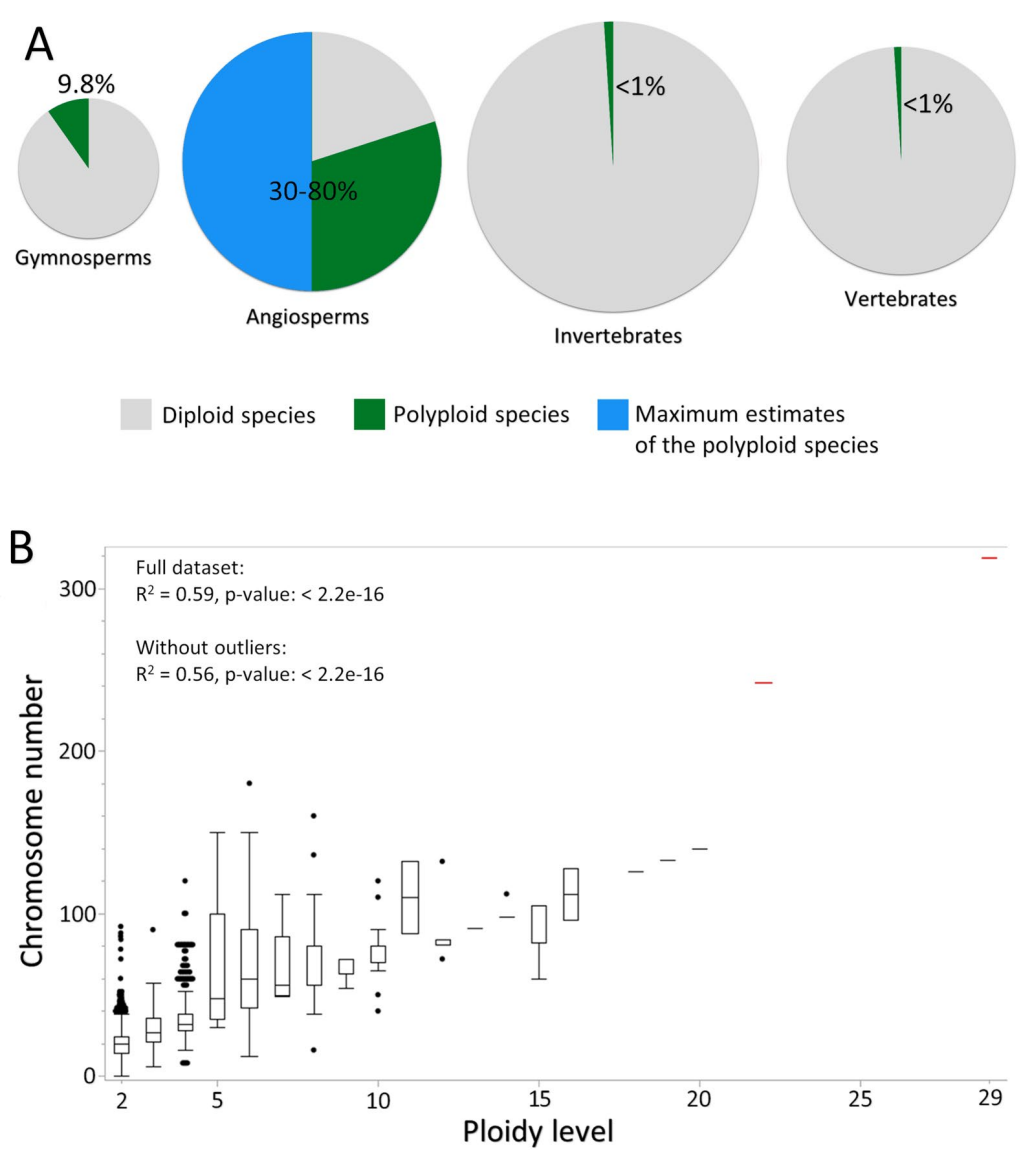
Occurrence of ploidy. **A**—percentage of polyploids (green and blue colours) and diploids (grey colour) in plants and animals; the size of the chart is proportional to the logarithm of the number of species in each group. **B**—correlation between the ploidy level and chromosome number in the plants; data according to the plant RNA database [44], plot prepared in JMP© software. R2 and p-value calculated using function lm in the R environment (R Core Team. 2021. R: A language and environment for statistical. https://www.R-project.org/) for two datasets: full and without outliers (red lines)

Additionally polyploidy may further provide genome stability and resistance to deleterious genetic mutations; for example, if a mutation occurs in one set of genes, another gene copy remains functional [7, 11]. Some phenotypic traits may undergo modifications related to the level of polyploidy. In the case of tetraploids (4n) and higher degrees of polyploidy, the amount of DNA and the number of chromosomes are even greater, which significantly impacts genome length (Fig. 2). This is a common phenomenon in the plant world. Although polyploidy can occur in some animal species, such as fish or insect species, it is much rarer and has a less significant impact on biodiversity among animals than plants. Polyploidy leads to a doubling or tripling, etc. of chromosome sets in plant cells. Each additional set of chromosomes contributes to the amount of DNA in the genome. As a result, the genomes of polyploids are larger. Polyploidy is associated with gene duplication. Each set of chromosomes can contain duplicate genes, leading to an increase in the number of gene copies in the genome, which can lead to greater genetic and functional diversity in the plant. Approximately 9.8% of gymnosperm species are polyploids [15], whereas this percentage varies for the angiosperm group, with estimates ranging between 30 and 80% [16]. However, it is possible that all seed plants were derived from a polyploid ancestor [17], and polyploidization was crucial for the evolutionary success of this group of plants. Genome duplication may occur multiple times within a single phylogenetic line, leading to the creation of large genomes. For example, the mulberry *Morus nigra* L. is a tetratetracontaploid (44x) and has 308 chromosomes [18]. However, there are plants with even larger genomes. The fern fern *Ophioglossum reticulatum* L., for instance, can be a decaploid with 1260 chromosomes [19]. The octoploid *Paris japonica* Franchet has one of the largest genomes, with a size of 148.8 Gb [20]. On the other hand, in addition to polyploids, which can have large genomes, diploid organisms may also possess extensive noncoding sequences that significantly increase genome length.

### The C- and G-value paradox

Genome length is associated with greater precision in the regulation of gene expression, which also affects adaptation to environmental conditions in plants [21–23]. The length of the plant genome may also be affected by the C- and G-value paradox [24–26]. This paradox arises from the correlation among genome size (C-value), the number of genes (G-value), and organismal complexity [25]. The G-value paradox was created to explain the apparent discrepancy between the number of protein-coding genes and organismal complexity [27]. The lack of correlation between genome size and the intuitively perceived complexity of an organism has been called the C-value paradox [28].

Another issue is the dynamics of the genome [29] as, in addition to polyploidy, the genome also undergoes epigenetic changes and variations related to the life cycle organisms.

Such a high level of genome complexity, especially in plants, poses challenges for researchers to precisely decode the information they contain, including both coding and noncoding sequences. Noncoding RNAs, such as lncRNAs, often have specific expression patterns related to molecular processes that determine what proteins should be made, when, and which genes should be turned on and off. However, the exact mechanisms of action are not well understood, especially in plant cells. This, in turn, is related to the difficulty of precisely identifying the sequences of various RNAs that perform their functions in the cell.

### Epitranscriptome

The complexity of the genome is also associated with long non-coding RNAs (lncRNAs), which have a significant impact on the epitranscriptome—the set of chemical modifications of RNA that regulate various aspects of RNA biology. How do lncRNAs influence the epitranscriptome? 1. Regulation of RNA methylation—lncRNAs can interact with RNA methyltransferases, such as METTL3 and METTL14, which catalyze the methylation of adenosine at position 6 (m6A) in mRNA [31, 32]. An example is the lncRNA XIST, which recruits the m6A complex, crucial for X chromosome inactivation [33]; lncRNAs can also influence RNA demethylation by interacting with demethylases such as FTO and ALKBH5, modulating m6A levels. 2. Interactions with RNA-binding proteins (RBPs)—lncRNAs can recruit RBPs that play key roles in RNA modification. They can attract proteins responsible for RNA editing, such as ADAR, affecting changes in RNA sequences through the deamination of adenosine to inosine [34–36]. 3. Chromatin structure and transcription modification—lncRNAs can influence structural modifications of chromatin, which in turn affect the transcription of protein-coding genes involved in the epitranscriptome. An example is the lncRNA HOTAIR, which recruits the PRC2 (Polycomb Repressive Complex 2), leading to histone modifications and transcriptional repression [35, 37]. 4. Stabilization or degradation of RNA—lncRNAs can affect the stability of other RNA transcripts by forming heteroduplexes with mRNA, which can lead to protection of mRNA from degradation or, conversely, targeting it for degradation by enzymes such as RNases [38, 39]. 5. Alternative splicing—lncRNAs can modulate mRNA splicing by influencing the selection of splice sites and the creation of alternative protein isoforms. An example is the lncRNA MALAT1, which interacts with SR proteins, regulating pre-mRNA splicing [40–42]. 6. Regulation of RNA transport—lncRNAs can affect the subcellular localization of mRNA, which is important for the efficiency of translation and mRNA stability. For instance, lncRNAs can direct the transport of mRNA to specific regions of the cell, such as sites of active translation [42, 43].

Through these mechanisms, lncRNAs have a broad and diverse impact on the epitranscriptome, modulating various aspects of RNA biology and influencing gene regulation at the post-transcriptional level.

The role and functions of lncRNAs are also highly complex in human and animal genomes. However, for plant genomes, not only are the functions of lncRNAs important for their better analysis, but there are also many gaps in knowledge about them. Expanded knowledge would facilitate understanding the functioning of entire plant genomes. In the case of plants, the possibilities of reliable analysis of the entire genome of a given species become complicated when we consider the overall genomic architecture including genome length, polyploidy, and the C-value and G-value paradox. 

### Model organisms and forest trees in plant lncRNA research

**Fig. 3:**
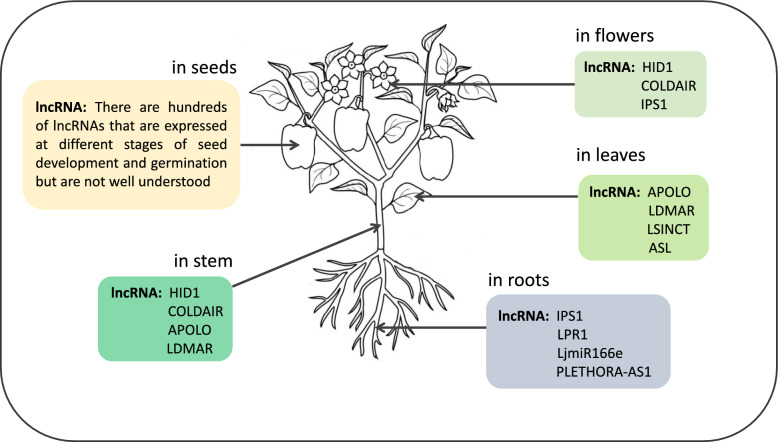
Examples of the action of long ncRNAs in different anatomical parts of the plant

Through high-throughput DNA sequencing and microarray technology, a vast number of lncRNAs have been identified, but only a small fraction of them have been characterized in terms of their functions [45] (Tables 1**, **2**, **Fig. 3). In eukaryotes, most of the genome is transcribed into noncoding RNAs (ncRNAs), which include long noncoding RNAs (lncRNAs). It is also known that lncRNAs play important roles in plant development and stress responses. However, the mechanisms and functions of lncRNAs may differ from those of other noncoding RNAs [46].

**Table 1:** Long noncoding RNAs and their involvement in model plant development

lncRNA	species	function	references
*Enod40*	*Medicago sativa ssp. varia*	Organogenesis	Crespi et al. [47]
*AtR8*	*Arabidopsis thaliana*	Hypoxic stress; defense mechanisms	Wu et al.[60]; Li et al. [61]
*MAS (Antisense RNA MAF4)* and *COOLAIR, COLDAIR, COLDWRAP,* *ASL (Antisense Long)*	*Arabidopsis thaliana*	Flowering	Zhao et al. [56]; Prall and Gregory [49]; Hung et al. [48]; Kim et al.[55]; Shin and Chekanova [54];
*asDOG1, 1GOD*	*Arabidopsis thaliana*	Seed germination	Fedak et al. [53]
*APOLO*	*Arabidopsis thaliana*	Auxin signaling	Wu et al. [52]
*HID1* *(Hidden Treasure 1)*	*Arabidopsis thaliana*	Light	Wang et al. [51]
*ELENA1* *(Elf18-Induced Long Non-Coding RNA1)*	*Arabidopsis thaliana*	Biotic stress	Seo et al. [39]
*TPSI, T5120, DRIR*	*Arabidopsis thaliana*	Abiotic stress	Franco-Zorrilla et al. 2007; Liu et al. [64]; Qin et al. [62];
*SVALKA*	*Arabidopsis thaliana*	Heat stress	Kindgren et al. [58]
asHSFB2a	*Arabidopsis thaliana*	Heat stress	Wunderlich et al. [67]
*FLORE (CDF5 Long Non-Coding RNA)*	*Arabidopsis thaliana*	Flowering	Henriques et al. [58]
*FLINC (Flowering Long Intergenic Non-Coding RNA)*	*Arabidopsis thaliana*	Flowering	Severing et al. [59]
*LINC-AP2* *(Long Intergenic Non-Coding RNA—APETALA2)*	*Arabidopsis thaliana*	Growth of the floral reproductive organs	Gao et al. [57]
*GARR* *(Gibberellin-Responsive lncRNAs)*	*Zea mays*	GA pathway	Li et al. [69]
DElncRNA	*Zea mays*	Heat stress	Hu et al. 2022
TE-lncRNA	*Zea mays*	Heat stress	Lv et al. [70]
*LAIR* (*LRK Antisense Intergenic RNA*)	*Oryza sativa*		Wang et al. [76]
*PMS1T* *(Photoperiod-Sensitive Genic Male Sterility 1)*	*Oryza sativa*	Flowering	Fan et al. [43]
*LDMAR* *(Long-Day Specific Male-Fertility-Associated RNA)*	*Oryza sativa*	Flower development and reproduction	Ding, Shen et al. [74]; Ding, Lu et al. [75]
*Ef-cd* *(Early Flowering-Completely Dominant)*	*Oryza sativa*	Flower development and reproduction	Fang et al. [77]
*lncRNA TCONS_00021861*	*Oryza sativa*	Heat stress	Chen et al. [78]
*lncRNA TCONS_00023703*	*Oryza sativa*	Seed development	Zhao et al. [121]

**Table 2:** Location and action of long noncoding RNAs in plants

Location of lncRNAs	lncRNA examples
lncRNAs in flowers	*COOLAIR* is a lncRNA involved in the regulation of flowering time in *Arabidopsis* (repressor of the FLOWERING LOCUS C gene, which inhibits flowering). COOLAIR helps maintain FLC in a repressed state, allowing the plant to flower *IPS1* (Induced by Phosphate Starvation 1) is a lncRNA involved in phosphate homeostasis and flowering in *Arabidopsis*. IPS1 acts as a decoy molecule for miR399. By sequestering miR399, IPS1 indirectly regulates PHO2 expression, which affects flowering time *COLDAIR* is a lncRNA that regulates the vernalization process in plants. COLDAIR is involved in the epigenetic regulation of FLCs. It helps establish and maintain a repressive chromatin state at the FLC locus during vernalization, enabling subsequent flowering *HID1* (HIDDEN TREASURE 1) is a lncRNA involved in floral development in rice. It regulates floral organ identity by modulating the expression of key genes. HID1 acts as a scaffold for the recruitment of chromatin-modifying enzymes, affecting the epigenetic regulation of floral genes
lncRNAs in leaves	*APOLO* (Auxin-Regulated Promoter Loop lncRNA) is a lncRNA involved in leaf development in *Arabidopsis thaliana*. It is regulated by auxin, a plant hormone that plays a key role in leaf development. APOLO acts as a scaffold for the assembly of a protein complex that promotes leaf development by regulating the expression of key genes involved in leaf morphogenesis *LDMAR* (Leaf Development Modulated by Abscisic acid-Responsive lncRNA) is a lncRNA discovered in rice that is responsive to abscisic acid (ABA), a hormone involved in various plant processes, including leaf development and stress responses. LDMAR regulates leaf shape by interacting with and modulating the activity of the transcription factor *Oryza sativa* ABA Responsive Element Binding Factor 3 (OsABF3) Leaf Senescence-Induced lncRNA (LSINCT) is a lncRNA associated with leaf senescence, programmed leaf senescence and leaf degradation. LSINCT is upregulated during leaf senescence in *Arabidopsis* and acts as a positive regulator of this process. It interacts with a transcription factor called WRKY75 to promote the expression of genes involved in leaf aging *ASL* (Asymmetric Leaf lncRNA) is a lncRNA involved in leaf polarity and asymmetric leaf development in tomato (*Solanum lycopersicum*). ASL regulates the expression of key genes involved in leaf polarity formation and growth, contributing to the formation of compound leaves with distinct leaflets
lncRNAs in stem	*HID1* is also involved in rice stem development. It regulates stem elongation by modulating the expression of genes involved in cell elongation and division. It is a scaffold for the recruitment of chromatin-modifying enzymes, affecting the epigenetic regulation of stem-related genes *APOLO* is also involved in stem development in *Arabidopsis thaliana*. It regulates stem elongation and growth by interacting with proteins involved in cell division and expansion. It helps coordinate the expression of genes involved in stem development and contributes to normal stem architecture *COOLAIR* acts as a regulator of FLOWERING LOCUS C (FLC), a gene that also affects stem elongation. COOLAIR helps maintain FLC in a repressed state, enabling normal stem growth and development *LDMAR* regulates stem elongation by interacting with and modulating the activity of the transcription factor *Oryza sativa* ABA Responsive Element Binding Factor 3 (OsABF3)
lncRNAs in roots	*LjmiR166e-3p-targeted* lncRNA was identified in *Lotus japonicus*, a legume. It acts as a target mimetic for microRNA166e-3p (LjmiR166e-3p) and plays a role in root development. By sequestering LjmiR166e-3p, lncRNA helps modulate the expression of genes involved in root growth and development *IPS1* is also involved in root development and nutrient uptake. IPS1 acts as a decoy molecule for miR399, a microRNA involved in phosphate homeostasis. By interacting with miR399, IPS1 indirectly influences the expression of genes involved in phosphate uptake and transport, affecting root growth and nutrient acquisition *LPR1* (Long Primaries Root 1) is a lncRNA discovered in *Arabidopsis* that regulates primary root growth. It works by modulating levels of auxin, a hormone crucial for root development. It is a scaffold for the assembly of a protein complex that regulates AUX/IAA protein degradation, thereby controlling auxin signaling and root growth *PLETHORA-AS1* is an antisense lncRNA that regulates the expression of PLETHORA genes, which are important regulators of root development in *Arabidopsis*. PLETHORA-AS1 acts by interacting with the chromatin remodeling protein PICKLE (PKL) to modulate PLETHORA gene expression and affect root growth and patterning
lncRNAs in seeds	Despite limited research on lncRNAs in seeds, hundreds of lncRNAs are expressed at different stages of seed development and germination. lncRNAs exhibit tissue-specific expression patterns and are potentially involved in the regulation of gene expression during seed development and germination. The researchers suggest that lncRNAs may be involved in the regulation of seed dormancy and may serve as potential targets to manipulate seed germination in crops. lncRNAs may play a role in the complex regulation of genetic imprinting during endosperm development and likely function in regulating lipid metabolism in seeds

The first lncRNA identified in plants is involved in organogenesis. Enod40, which induces changes in the subcellular localization of nuclear RNA-binding proteins, was isolated from *Medicago sativa* ssp. *varia* [47].

The flowering-associated lncRNA *COLDAIR*, discovered in *Arabidopsis thaliana* L., acts by remodeling chromatin and altering the expression of the *FLOWERING LOCUS C* (*FLC*) gene, which is the most important regulator of flowering, repressing the expression of the *FLOWERING LOCUS* T (*FT*) integrator [48]. *COOLAIR* and *COLDAIR* are well established in *Arabidopsis thaliana* [46, 48, 49], a model plant for studying the function of all lncRNAs [50]. In *Arabidopsis*, lncRNAs that play a role in processes related to plant development, such as light (*HID1*) [51] and auxin signaling (*APOLO*) [52] have been identified. Additionally, lncRNAs involved in seed dormancy (*asDOG1, 1GOD*) [53] and flowering (*COOLAIR, COLDAIR, COLDWRAP, ASL* and *MAS*) [48, 54–56] or flower development and reproduction (*FLORE, FLINC, LINC-AP2*) [57–59] have also been reported. The lncRNA *AtR8* is transcribed by RNA polymerase (Pol III), associated with hypoxic stress, and involved in defense mechanisms [60, 61]. One of the most important functions in plants is the response to biotic (*ELENA1*) [39] and abiotic stresses (*TPSI lncRNA, T5120, DRIR, SVALKA*) [62–66]. Plants do not have adaptive immunity. Thus, in the case of biotic stress, they counteract pathogens via an innate immune system, pattern-induced immunity (PTI), and lncRNAs modulate PTI in *Arabidopsis* [50]. In the case of abiotic stress, lncRNAs help to combat or adapt to it. This is the case for *SVALKA* and the long noncoding antisense RNA *asHSFB2a* [67]. Transcriptional heat stress factors (HSFs) are the most important regulators of the heat stress response. Many lncRNAs are induced by stress, but it is most likely that not all of these lncRNAs have regulatory functions. As discovered in *Arabidopsis*, lncRNAs also function in the RNA-dependent DNA methylation (RdDM) pathway, the main function of which is to silence transposons and maintain genome integrity [50].

The transcriptome of maize, *Zea mays* L., contains lncRNAs with a single exon and is distinct from that of protein-coding transcripts, with a large proportion being intergenic lncRNAs [68]. Most lncRNAs exhibit evolutionary variation and are specific to the tissue and developmental stages of the plant, indicating functional divergence of lncRNAs across the genome. Some lncRNAs, such as GARRs, are expressed and modulate the response to gibberellin (GA), a hormone essential for growth and development [69], and likely regulate transcription in the GA pathway [68]. Transposable elements (TE) can contribute to the formation of lncRNAs in maize, and transposon-derived lncRNAs and TE-lncRNAs respond to abiotic stresses (heat, drought, cold, salt) [70]. LncRNAs (differentially expressed, DElncRNAs) also play important roles in the response to heat stress, which affects maize development, particularly under excessive ambient temperature. In response to heat stress, thousands of transcripts, including noncoding RNAs (miRNAs and lncRNAs) as well as protein-coding genes, function as regulators, controlling the expression of target genes [71]. Thus, identifying as many target genes as possible is one of the primary goals for understanding the biological functions of lncRNAs. The high temperature to which a plant is subjected can cause oxidative stress and the production of excessive amounts of reactive oxygen species (ROS), which can also affect hormone production, further influencing lncRNA activity. Maize plants are sensitive to salt stress, and hundreds of millions of hectares of land worldwide are affected by salinity, resulting in decreased maize yields. Like heat stress, salt stress leads to increased ROS and oxidative stress. In maize seedlings, lncRNAs affect the molecular regulatory networks of genes responsible for combating salt stress. Studies conducted on salt stress in maize have shown considerably higher expression of lncRNAs compared with protein-coding genes [72].

In rice *Oryza sativa* L., the *PMS1* (photoperiod-sensitive male sterility locus 1) gene is associated with photoperiod-sensitive male sterility (PSMS) and encodes the long noncoding RNA PMS1T, which is expressed in young panicles as a regulator of pollen development [73]. The locus regulating PSMS (*pms3*) encodes the lncRNA *LDMAR*, which is associated with normal male fertility in rice [74]. Mutants characterized by PSMS have contributed significantly to the development of hybrids (hybrid rice), the cultivation of which has resulted in a global increase in rice productivity [75]. The lncRNA *LAIR* plays a role in the regulation of rice yields, increasing them [76]. It is transcribed from the antisense strand of the leucine-rich repeat receptor kinase (LRK) gene cluster, named *LAIR* (LRK antisense intergenic RNA), and *LAIR* overexpression regulates the expression of several LRK genes [76]. Overexpression of *LAIR* in transgenic rice lines leads to the production of more primary panicles and more panicles per plant overall, resulting in better plant growth. Therefore, the authors of this study suggest that the lncRNA LAIR may contribute to improved crop yield in general [76].

Another lncRNA in rice is the lncRNA Ff-cd, the expression of which reduces maturation time while maintaining yield [77]. The lncRNA Ef-cd is a long noncoding RNA transcribed from the antisense strand of the OsSOC1 flowering activator locus that can positively regulate its expression, improve the rate of photosynthesis and contribute to easier nitrogen utilization from the soil [77]. This activity of the lncRNA Ef-cd contributes to improving the adaptability of the plant; thus, according to the authors of these studies, it may improve extensive rice cultivation. Plants adapt to stress by modulating signaling pathways in which lncRNAs play an important role. An example is *TCONS_00021861*, a long rice noncoding RNA associated with drought tolerance. The lncRNA *TCONS_00021861*, through interaction with miR528-3p, regulates the *YUCCA7* gene [78]. *YUCCA* (*YUC*) genes are associated with plant drought resistance [79–81]. Overexpression of the lncRNA *TCONS_00021861* in a plant subjected to drought stress slows growth reduction and reduces ROS accumulation [78].

Long noncoding RNAs involved in the abiotic stress response are also known to be involved in the abiotic stress response of wheat *Triticum aestivum* L. and cotton *Gossypium* spp. In wheat, these RNAs interact with miRNAs (miR398), similar to that noted in rice. Overexpression of three lncRNAs (lncR9A, lncR117 and lncR616) in transgenic plants increases cold resistance [82]. In contrast, lncRNA 973 overexpression in transgenic cotton plants enhances drought tolerance [83].

The identification of lncRNAs and their functions in trees has been reported for very few species, including *Eucalyptus grandis* W. Hill ex Maiden [84], Chinese white poplar *Populus tomentosa* Carrière [85], *Paulownia* spp. [86] and the tung tree *Vernicia fordii* Hemsl. [87].

There is virtually no research on lncRNAs and their functions during adaptation processes in the context of climate change or aging seeds. Analyses of lncRNAs expressed under cold stress or in stored seeds have not been performed. Both of these topics are of great importance to forestry. First, some tree species, such as Norway spruce *Picea abies* (L.) H. Karst, are particularly vulnerable to the effects of climate change, including prolonged droughts, extreme temperatures, and increased pest outbreaks [88–90]. Understanding the role of lncRNAs in these adaptation processes could provide critical insights into improving the resilience of forest ecosystems. The second topic is related to seed storage, afforestation, the use of high-quality seed material, and the aging of seeds and endangered species [91]. Nevertheless, we know that lncRNAs are involved in the response to high- and low-temperature stress in other organisms [92–94]. In addition, during seed development, lncRNAs also exhibit different expression patterns, the main influence being RNA interference by *TCONS_00023703* [95]. A number of aging-related lncRNAs have also been shown to play important roles in the cell in regulatory processes at both the transcriptional and translational stages, as well as at the posttranscriptional and posttranslational stages [45, 96, 97].

It is crucial to undertake further research to investigate the role of lncRNAs in forest tree species, particularly in the context of climate change. For example, Norway spruce, a species of significant economic and ecological importance, has been introduced to regions beyond its natural range due to its fast growth and high productivity [98, 99]. However, the artificial planting of spruce monocultures outside optimal ecological conditions has reduced their resistance to biotic and abiotic stressors, making them particularly vulnerable to climate change [100–104].

**Fig. 4:**
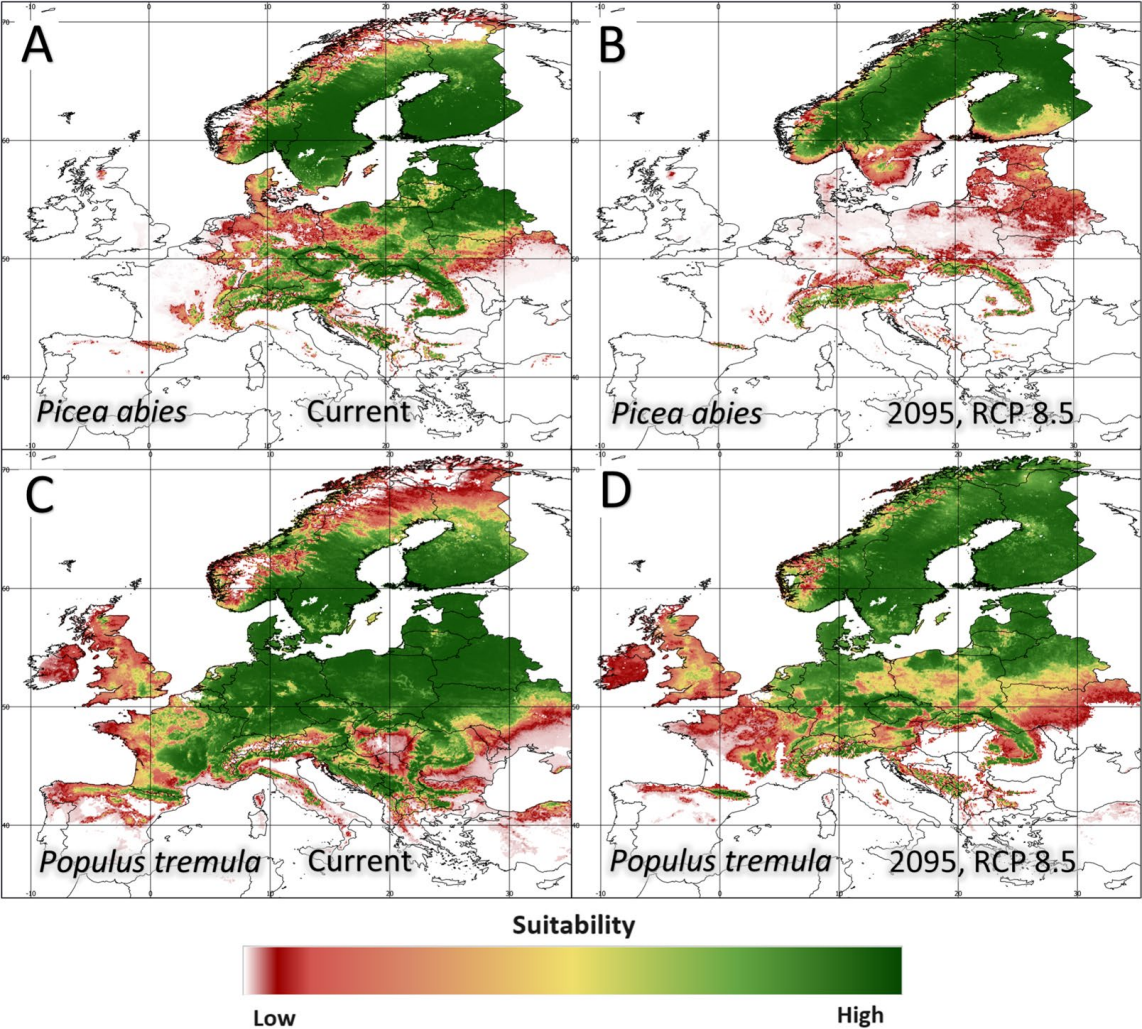
Changing of the potential range of two woody species according to [111]. **A**—Potential range of Picea abies in current conditions; **B**—Potential range of Picea abies in 2095 (RCP 8.5), **C**—Potential range of Populus tremula in current conditions; **D**—Potential range of Populus tremula in 2095 (RCP 8.5). Maps were prepared using QGIS software (QGIS Geographic Information System. QGIS Association. 2023. http://www.qgis.org)

Increasing climate change results in extreme and marginal weather conditions (e.g., prolonged spring droughts, high temperatures in summer, lack of snow cover in winter, and excess heavy rains or storms with destructive winds) and disturbances in soil conditions (e.g., decreased groundwater availability and an increase in evapotranspiration). Such phenomena decrease the health conditions of trees and increase stress and susceptibility to secondary pests (e.g., bark beetle outbreaks). The interaction of these threats results in increased mortality of Norway spruce stands [99, 105, 106]. In extreme cases, the dieback of large fragments of forest stands is observed, forcing foresters to change species composition and remove Norway spruce from stands [107–109]. Spruce clearly cannot cope with the climate events currently occurring (Fig. 4). However, the problem concerns not only spruce but also other forest trees [110], which also struggle with adaptation difficulties due to changes in the environment (Fig. 4).

Attempts to predict the impact of climate disturbance on the ranges of forest tree species (including *Picea abies*) indicate significant changes in Europe over the next few decades [112]. Attention should be given to the broader consequences of changes in tree species distributions, which include alterations in the species composition of tree stands and, consequently, changes in the biodiversity of all forest ecosystems [113]. Understanding the mechanism of species and population self-defense at the molecular level, including that related to lncRNAs, may enrich plans for the assisted migration of forest tree species. In this regard, setting a clear and coherent course of action, both geographically and ecologically, for the coming years seems to be the most urgent topic [114]. Observations in provenance experiments have allowed us to learn about the variability and plasticity of tree populations at the level of morphological and physiological markers [99, 115]. However, existing knowledge should be supplemented with detailed analyses at the molecular level. This will facilitate an understanding of the mechanisms of adaptation to changing forest tree life conditions. Noncoding protein genes play a key role in controlling the regulation of gene expression under abiotic stress conditions [116].

The potential of noncoding RNAs to induce chromatin changes that can be epigenetically inherited suggests that they may also play a significant role in plant phenotypic plasticity to environmental changes [117–119]. Addressing this issue is particularly important in the face of global warming and regional climate fluctuations, which are causing increased drought stress in forest ecosystems. Climate projections for Central Europe predict an increase in temperature and a decrease in rainfall in summer, which is already noticeable. In the future, a warmer climate with drier summers may cause these changes to increasingly negatively impact the growth of spruce and other trees that cannot cope with drought [90]. The impacts and threats posed to spruce by a changing climate may vary by region, with more severe impacts expected in regions where drought causes physiological stress on spruce [88]. Monitoring changes in the long noncoding RNA transcriptome in spruce seeds, that exhibited a low adaptation to climate change [88–90], seems important in the context of the accumulated knowledge.

Populations that can adapt to climate change produce seeds with greater survival potential, which has implications for the overall suitability of seeds but is also important for storing them for use in commercial forests. Plant lncRNAs are known to be associated with plant growth and development and play major roles in modulating environmental responses to biotic and abiotic stresses [119–121]. However, due to the versatility of plant lncRNAs and their heterogeneity, we have limited knowledge about them. Most of them, unlike mammalian lncRNAs, remain unexplored.

### Transcriptomic regulation using antisense lncRNAs

**Fig. 5:**
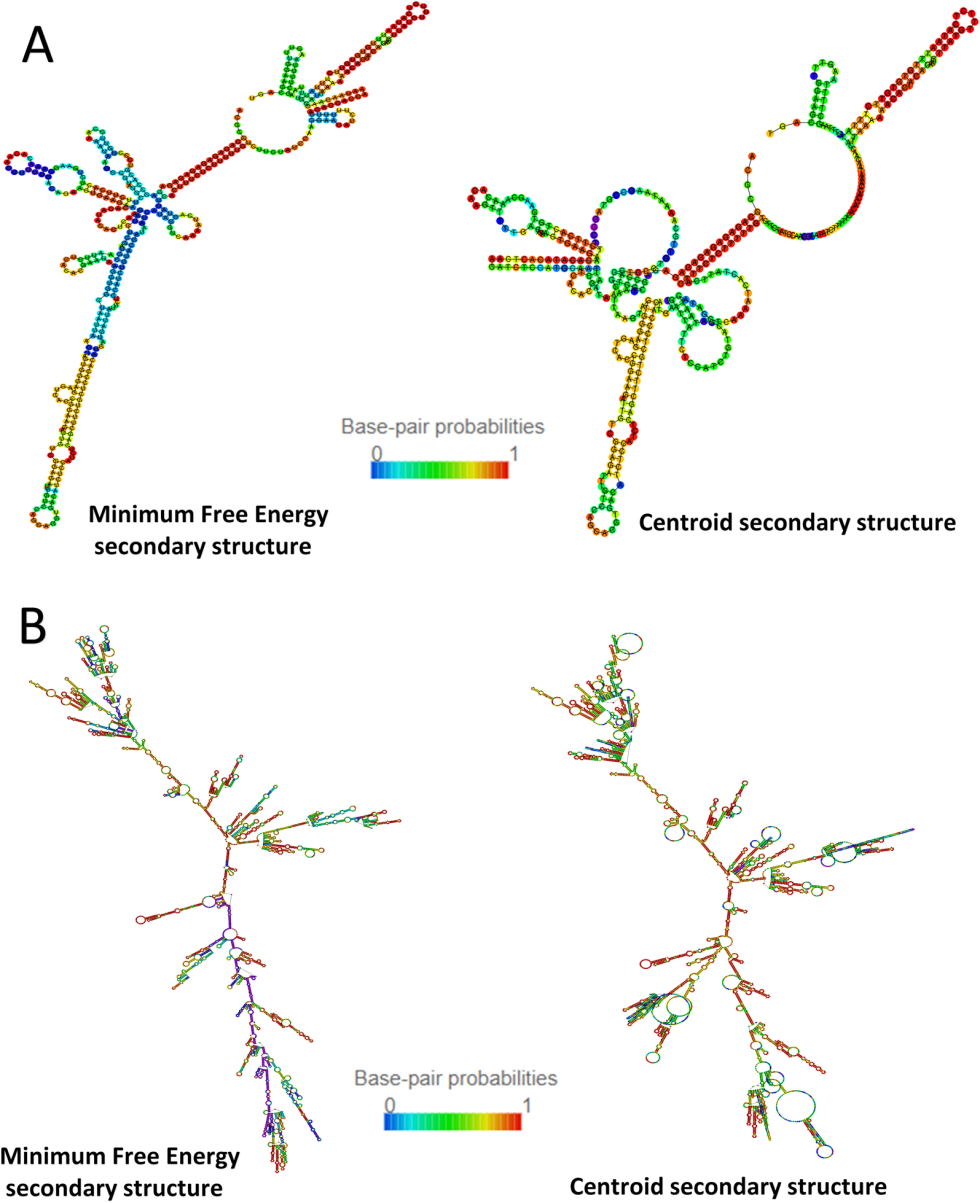
Minimum Free Energy (MFE) and Centroid secondary structures for the lncRNA and its target: **A**—COOLAIR lncRNA (full length, noncoding primary transcript from TAIR database (The Arabidopsis Information Resource, on www.arabidopsis.org, 08.03.2024); accession number: 6533802487, name: **A**T5G01675.1). **B**—FLOWERING LOCUS C gene, sequence according to NCBI database: NC_003076.8. Structures were prepared using the RNAfold tool [142, 143]

An antisense lncRNA is a long noncoding RNA (lncRNA) that is complementary to a ribonucleic acid (usually mRNA) sequence [122]. It can interact with complementary RNA sequences by forming double-stranded RNA‒RNA structures. The names of antisense lncRNAs refer to the recognized trait or function: antisense IGF2R nonprotein-coding RNA (AIRN), antisense HOX antigenic RNA (HOTAIR), COOLAIR (its expression increases rapidly during early vernalization in plants) (Fig. 5) or auxin-related signaling (APOLO) [72, 123, 124].

In humans, antisense lncRNAs play regulatory roles in cancer [72] and are also highly important in plants, where they can regulate gene expression by interacting with complementary mRNA sequences [125]. Antisense lncRNAs can inhibit gene transcription by interfering with the transcription process [126]. They can also bind to mRNAs, either stabilizing them or accelerating their degradation [127]. This directly influences the availability of mRNA for a given genetic sequence. Antisense lncRNAs can affect alternative splicing of pre-mRNAs, thereby affecting the diversity of protein isoforms in the cell. They can also affect the cellular localization of mRNAs, controlling the transport of mRNAs between the nucleus and cytoplasm. By binding to mRNAs, antisense lncRNAs can control the translation process, affecting the ability of mRNAs to act as a matrix for synthesized proteins. Additionally, they can affect the stability of proteins by controlling their degradation or interacting with mRNA molecules encoding these proteins [125].

The sites of action of antisense lncRNAs in plants may vary. Antisense lncRNAs (aslncRNAs) can interact with complementary RNA sequences in the cell nucleus, affecting transcriptional processes, alternative splicing, or the cellular localization of mRNAs. They can also influence mRNAs in the cytoplasm, controlling translation or mRNA stability [127]. Additionally, they can regulate the cellular transport of mRNAs. lncRNAs can form complexes with mRNAs within various ribonucleoprotein (RNP) structures, significantly impacting mRNA stability, translational processes, and mRNA localization [124]. Moreover, antisense lncRNAs can affect ribosomes, the site of mRNA translation into proteins, influencing the translation process and controlling mRNA availability to ribosomes. They can bind to a variety of cellular structures and organelles, such as plastids, mitochondria, and the endoplasmic reticulum, affecting biological processes in these structures [128]. The interaction of antisense lncRNAs with mRNAs and other RNA molecules plays a crucial role in the regulation of gene expression and cellular functions in plants [72, 129].

Antisense lncRNAs play a crucial role in gene regulation in plants, particularly under stress conditions [64, 78, 129–131]. Understanding their mechanism is essential for elucidating key biological processes in plant cells and uncovering their functional roles in plant biology.

The formation of antisense RNA is linked to gene expression. lncRNAs can regulate the expression of neighboring coding genes (cis-regulation) and the expression of genes on different chromosomes (trans-regulation). When a gene is ready to produce the protein it encodes, the two strands of DNA unravel. The first strand, the 'coding' strand, is 'transcribed', and a molecule of messenger RNA (mRNA) is formed, which serves as a template for protein production. The second DNA strand does not contain the information needed to produce the protein; nevertheless, an RNA is occasionally produced from it—the so-called 'antisense' RNA—with a sequence complementary to the mRNA. If the mRNA binds to the antisense RNA, it is blocked, and no protein is synthesized. Many antisense RNAs have been identified for many genes. However, until recently, their functions were unknown, and they were assumed to be uniquely associated with gene expression. Given the multitude of genes for which antisense RNA is produced, it is possible that it represents a new, hitherto unrecognized step in the regulation of gene expression. Many noncoding transcripts have regulatory functions, but the sheer number of transcripts across the genome may be underestimated because transcripts from the antisense strand of protein-coding genes are often rapidly degraded [132].

A strong source of antisense transcription is transcription terminators (at the 3′ end of genes) [133]. However, bidirectional gene transcription in two orientations on opposite strands or bidirectional promoters has been cited as the main cause of antisense transcription [132, 134]. In animals, we have a nuclear and a mitochondrial genome, but plants also have a chloroplast genome, which is also involved in significant bidirectional transcription. Transcripts associated with basic biological processes, such as photosynthesis or metabolism, are more stable in plants than are transcripts associated with the regulation of gene expression responsible for signal transduction, hormones or response to stimuli [135, 136].

Although fewer in number, antisense transcripts are not necessarily less important. They can induce chromatin changes through histone turnover, a process crucial for gene regulation [132]. Studies have shown that histone modifications, antisense transcription and histone turnover are highly correlated. Specifically, downregulation of antisense transcripts results in changes to histone features, such as increased H3 acetylation but decreased H3K36me3 [132].

An example of the relationship between antisense transcription and gene regulation is the *DOG1* gene, which is associated with seed dormancy. DOG1 is regulated by the antisense transcription of lncRNAs (otherwise known as *asDOG1* or *1GOD*), which represses gene expression during normal seed growth [137]. In this case, antisense transcription is induced by abscisic acid (ABA) and drought. Transcription of *asDOG1* originates from the 3′ end of DOG1 near the major poly(A) site. The expression of the DOG1 mRNA isoform occurs most strongly in seeds, and expression of the DOG1 antisense transcript (asDOG1) is highest in seedlings [137]. Antisense transcription negatively regulates both *DOG1* expression and seed dormancy [53]. Researchers have shown that cold-induced *FLC* antisense transcripts play a role in the epigenetic silencing of the *FLC* gene in *Arabidopsis* [138]. There are more examples of antisense transcript activity in plants, such as the natural antisense transcript *NAT-DONE40* locus ENOD40 [139] or a natural antisense transcript that reduces the expression of the drought stress-responsive gene *ZmNAC48* in maize [140] and NATs paired with mRNAs involved in the defense response [141].

LncRNAs have secondary structures that are often more conserved than their nucleotide sequences. These structures are believed to be crucial for their functionality, providing insight into the functional significance of lncRNAs. The major lncRNA COOLAIR variants are characterized by a complex, multidomain structure [144]. The conservation of COOLAIR’s secondary structure suggests that it plays a functional role beyond simple antisense transcription. The distally polyadenylated transcript undergoes complex folding, influenced by a single noncoding SNP that defines a functionally distinct *A. thaliana* FLC haplotype. The long noncoding RNA COOLAIR is not only essential for plant adaptation to environmental stresses [145], but also contributes significantly to gene regulation, vernalization and flowering responses through its complex secondary structure. Understanding these mechanisms enhances our knowledge of how plants adapt to changing environmental conditions, which is particularly relevant in the context of climate change.

### Are lncRNAs coexpressed in plant genomes?

Long noncoding RNAs (lncRNAs) play both direct and indirect regulatory roles in key biological processes, including development, vernalization, and adaptation to environmental stress. These highly versatile molecules interact with RNA, DNA, and proteins, forming complex regulatory networks. Some lncRNAs are closely associated with microRNAs (miRNAs), which can be derived from lncRNAs and function in both coding and noncoding transcripts. These interactions often involve intricate, incompletely understood regulatory mechanisms based on noncoding RNAs. Cytoplasmic lncRNAs, for example, modulate gene expression posttranscriptionally through miRNA-mediated interactions and by binding to cytoplasmic proteins [146].

In humans, many lncRNAs are coexpressed with mRNAs, and their interactions have been extensively studied [123, 147–149]. miRNA‒mRNA and mRNA‒lncRNA interactions are known to play critical roles in cancer [143, 144] and other diseases [150, 151] and other diseases [152]. In plants, lncRNAs are involved in immunity mediating plant-pathogen interactions and immune responses [153]. However, the molecular mechanisms and regulatory pathways underlying lncRNA coexpression in plants remain poorly understood. Studies have shown that miRNA-lncRNA pairs, which are coexpressed and stress-responsive, are differentially expressed under drought conditions [154]. For instance, the lncRNA TCONS_00021861 associated with the *YUCCA7* gene, modulates the level of miR528-3p, increasing indole-3-acetic acid (IAA) levels and enhancing drought tolerance [78]. In plants, some lncRNAs not only serve as targets or sources of miRNAs but also regulate the biogenesis and activity of miRNAs [155]. Certain lncRNA transcripts can be targeted by miRNAs, leading to the production of phased small interfering RNAs (siRNAs) [156]. Additionally, lncRNAs can influence pri-miRNA processing, act as miRNA mimics, or inhibit miRNA expression [156, 157]. Interactions between antisense lncRNAs and mRNAs also play roles in immunity and cell metabolism, with coexpressed lncRNAs regulating the expression of protein-coding genes [94, 158].

While lncRNAs are primarily known for their role in gene expression regulation, they also perform other functions, such as acting as scaffolds for proteins or other RNAs [146]. One notable example is the telomerase component lncRNA TR (also known as TER or TERC), which is part of the telomerase ribonucleoprotein complex (RNP) and is found in plants [159, 160]. This lncRNA is essential for maintaining chromosome ends and serves as a template for telomere replication through reverse transcription by telomerase. The lncRNA TR also acts as a scaffold for reverse transcriptase (TERT) and other accessory proteins [146].

### Perspectives

Expanding our understanding of lncRNAs in plants is critical due to their pivotal roles in gene expression regulation, adaptation to environmental conditions, and developmental processes. Research on lncRNAs has deepened our knowledge of gene regulatory mechanisms, including their impact on chromatin remodeling, mRNA stability, translation, and alternative splicing. Moreover, lncRNAs are integral to plant responses to abiotic and biotic stresses, making them essential for crop breeding and environmental protection. Discoveries related to lncRNA functions in processes such as flowering, germination, and reproductive organ formation have shed light on the molecular mechanisms controlling these events. Interspecies interactions involving lncRNAs represent a promising area of research with applications in agriculture, forestry, and plant ecology. Comparative studies of lncRNAs across plant species provide valuable insights into the evolution of plant genomes, particularly in forest trees. Further research on lncRNAs could lead to innovative methods for improving crop yields, enhancing stress resilience, and promoting sustainable resource management. In summary, lncRNA research is vital for both advancing fundamental biological knowledge and developing practical solutions for agriculture and environmental conservation.

Another emerging area is the role of the epitranscriptome in lncRNA biology. The epitranscriptome, which encompasses chemical modifications of RNA, influences various aspects of plant development, including stress responses and adaptations [161–163]. Advances in sequencing and RNA editing technologies have enabled a deeper understanding of the epitranscriptome, revealing over a hundred posttranscriptional modifications in mRNAs, rRNAs, tRNAs, and lncRNAs [162]. These discoveries are helping to elucidate how lncRNAs function and interact at the molecular level.

Despite significant progress, the characterization and identification of lncRNAs in plants remain imprecise. Current lncRNA annotations are limited, and more research is needed to fully understand their functions. Modern technologies, such as high-throughput sequencing and CRISPR, are crucial for uncovering the roles of lncRNAs. Additionally, traditional machine learning (ML) and deep learning (DL) models are being applied to lncRNA research, offering new ways to analyze their functions and evolution [164, 165]. However, developing accurate predictive models for lncRNAs is challenging, and ML/DL methodologies still face significant hurdles. In plants, lncRNAs play critical roles in developmental regulation, making it essential to understand their molecular mechanisms. Predictive tools, such as the hybrid deep learning model PlncRNA-Hdeep are emerging as valuable resources for improving lncRNA annotation and functional analysis [166]. Rapid advancements in artificial intelligence and computational analysis are accelerating the creation of large-scale, publicly available databases for plant lncRNAs, which will enhance our understanding of these molecules across diverse plant species, including forest trees.

Unlike human and animal genomes, which are generally more compact and structurally less variable, plant genomes show a high level of complexity. Many plant species have much larger and more repetitive genomes, making sequencing and annotation more challenging. For example, the wheat (*Triticum aestivum*) genome is about five times larger than the human genome and contains extensive duplications, complicating assembly and functional annotation. As a result, the development of comprehensive genomic databases for plants is lagging behind those for humans and animals, as plant genomes require more complex projects and therefore much more funding. Resources such as Phytozome, The Arabidopsis Information Resource (TAIR) and Gramene provide essential plant genomic data, but their depth and quality of annotation often lags behind their human and animal counterparts. The slower pace of plant genomic research is delaying the discovery of genes associated with traits such as stress resistance, disease susceptibility and yield improvement.

Differences in funding for genomic research contribute significantly to the different rates of progress in animal and plant genomics. The prioritisation of human and biomedical research results in well-funded, highly detailed genomic databases, while plant genomic research struggles with limited resources and technical challenges related to genome complexity. Addressing the funding imbalance is critical to accelerating plant genomic research, increasing agricultural productivity and ensuring food security in the face of climate change. Increased investment in plant genomics would enable the development of more comprehensive databases, supporting advances in plant breeding, biotechnology and environmental and forest sustainability.

## Conclusion

Compared to human and animal genomes, plant genomes are larger, often polyploid, and exhibit dynamic epigenetic modifications. These characteristics make plant genome research more complex, time-consuming, and costly. The intricate regulatory mechanisms involving lncRNAs further complicate the study of plant genomes. Advanced computational tools, including machine learning and deep learning, are indispensable for predicting and annotating lncRNAs, which remain challenging subjects due to the complexity of plant genomes. The study of lncRNAs in plants provides essential insights into gene regulation, stress responses, and adaptation mechanisms. lncRNAs, including antisense lncRNAs, regulate gene expression through diverse mechanisms, such as mRNA stabilization, translation modulation, and chromatin modification. Chromatin is critical for plant responses to biotic and abiotic stresses and play a key role in adaptation to changing environmental conditions.

Research on RNA modifications has opened new avenues for understanding lncRNA functionality, particularly their impact on the epitranscriptome. lncRNAs are involved in RNA methylation, chromatin remodeling, RNA stability, transport, and splicing, as well as interactions with RNA-binding proteins. Exploring the epitranscriptome offers a promising frontier for uncovering additional layers of lncRNA regulation.

lncRNA research has the potential to revolutionize plant biology, driving innovations in sustainable agriculture and environmental management. Understanding lncRNA functions in genomic networks could lead to strategies for enhancing crop resilience, mitigating environmental stresses, and conserving natural resources. Continued exploration of lncRNA mechanisms promises to unlock new approaches for plant sciences.

While much of our knowledge comes from model organisms like Arabidopsis thaliana, understanding lncRNAs in non-model species and forest trees is limited. These species face unique challenges, particularly in the context of climate change. Bridging this knowledge gap is essential for developing effective conservation strategies and improving crop resilience. Comprehensive and interdisciplinary research efforts, supported by advanced computational tools, are needed to fully decipher the role of lncRNAs in various plant species, including forest trees. Such efforts will enhance our understanding of gene regulatory networks and may contribute to the development of innovative solutions eg. for agriculture and environmental conservation, including forest ecosystems in an era of climate change. The study of lncRNAs in plant genomes, although difficult and very tedious, has great potential for advancing plant biology and addressing global challenges in plant science.

## Data Availability

Not applicable.
